# Estimating the contribution of a service delivery organisation to the national modern contraceptive prevalence rate: Marie Stopes International's Impact 2 model

**DOI:** 10.1186/1471-2458-13-S2-S5

**Published:** 2013-06-17

**Authors:** Michelle B Weinberger, Kenzo Fry, Tania Boler, Kristen Hopkins

**Affiliations:** 1 Marie Stopes International (MSI), London, W1T 6LP, UK

## Abstract

**Background:**

Individual family planning service delivery organisations currently rely on service provision data and couple-years of protection as health impact measures. Due to the substitution effect and the continuation of users of long-term methods, these metrics cannot estimate an organisation's contribution to the national modern contraceptive prevalence rate (CPR), the standard metric for measuring family planning programme impacts. Increasing CPR is essential for addressing the unmet need for family planning, a recognized global health priority. Current health impact estimation models cannot isolate the impact of an organisation in these efforts. Marie Stopes International designed the Impact 2 model to measure an organisation's contribution to increases in national CPR, as well as resulting health and demographic impacts. This paper aims to describe the methodology for modelling increasing national-level CPR as well as to discuss its benefits and limitations.

**Methods:**

Impact 2 converts service provision data into estimates of the number of family planning users, accounting for continuation among users of long-term methods and addressing the challenges of converting commodity distribution data of short-term methods into user numbers. These estimates, combined with the client profile and data on the organisation's previous year's CPR contribution, enable Impact 2 to estimate which clients maintain an organisation's baseline contribution, which ones fulfil population growth offsets, and ultimately, which ones increase CPR.

**Results:**

Illustrative results from Marie Stopes Madagascar show how Impact 2 can be used to estimate an organisation's contribution to national changes in the CPR.

**Conclusions:**

Impact 2 is a useful tool for service delivery organisations to move beyond cruder output measures to a better understanding of their role in meeting the global unmet need for family planning. By considering health impact from the perspective of an individual organisation, Impact 2 addresses gaps not met by other models for family planning service outcomes. Further, the model helps organisations improve service delivery by demonstrating that increases in the national CPR are not simply about expanding user numbers; rather, the type of user (e.g. adopters, provider changers) must be considered. Impact 2 can be downloaded at http://www.mariestopes.org/impact-2.

## Background

An estimated 222 million women in developing countries have an unmet need for modern contraceptives [1]. This deficit puts women at risk of unintended pregnancies, which can result in unsafe abortions or even maternal death. Such a high level of unmet need underscores the pressing global health problem of access to modern contraceptive services, an issue the wider reproductive health and donor community has recognised and prioritised. Recently, the July 2012 Family Planning Summit announced a commitment to reach 120 million additional women in the world's poorest countries with family planning services, in order to reduce the number of unintended pregnancies and improve maternal and child health [2]. Key benchmarks for global health also recognise this unmet need. Increasing contraceptive use is one of the chief indicators of Millennium Development Goal 5 which aims to improve maternal health [3]. Similarly, the Department for International Development's Framework for Results for improving reproductive, maternal, and newborn health monitors the number of additional modern family planning users reached, with the aim of creating at least 10 million more users by 2015 [4].

The modern contraceptive prevalence rate (CPR), a figure that represents the proportion of women of reproductive age (WRA) (15-49 years) using modern methods of contraception, serves as the key metric for measuring contraceptive uptake in the developing world [3]. To increase CPR, family planning programmes must augment the proportion of WRA using modern contraception, in addition to maintaining contraceptive use among existing family planning users. The modern CPR is comprised of women using short-term methods, such as oral contraceptive pills, condoms, and injectables, and long-acting and permanent methods (LAPMs), such as intrauterine devices (IUDs), implants, and sterilisation.

Ultimately, increases in CPR will be achieved through the joint efforts of a wide range of actors across the family planning sector and healthcare systems of developing countries, including public and private providers, product distributors, and family planning advocates. Marie Stopes International (MSI), a United Kingdom-based, international non-governmental organisation, plays a key role in these efforts. Operating in 42 countries throughout the world, MSI's extensive network of clinics, outreach teams, and social franchises strengthens health systems through its delivery of high-quality reproductive health services to underserved women, with an emphasis on clinic and provider capacity-building, modern contraceptive choice, and quality assurance. As part of its rigorous programme monitoring, the organisation places a priority on understanding its contribution towards the global goal of achieving universal access to contraception [5]. However, as discussed below, attributing national level increases in CPR to a single organisation - using a metric called "increasing CPR" - is inherently difficult due to two key challenges.

Both challenges derive from the metrics that service delivery organisations typically use to measure programme achievement. Most of these organisations track programme progress through their service delivery outputs, such as the number of commodities and services provided. Organisations also use couple-years of protection (CYPs), a metric that weights each family planning method according to its effectiveness and the duration of protection against pregnancy that the method provides to the couple. For example, one 10-year IUD equates to 4.6 CYPs, a figure based on the IUD's average duration of use, while one contraceptive pill cycle provides 1/15th of a CYP, since 15 pill cycles are needed for a full year of protection [6]. To derive the total number of CYPs generated by a programme in a year, service provision data by method distribution are multiplied by each of these factors. While service delivery outputs and CYPs are useful for understanding the scale of family planning programmes, they do not measure how these organisations contribute towards increasing CPR. As a result, service delivery outputs and CYPs cannot demonstrate an organisation's national level health impact.

**Figure 1:**
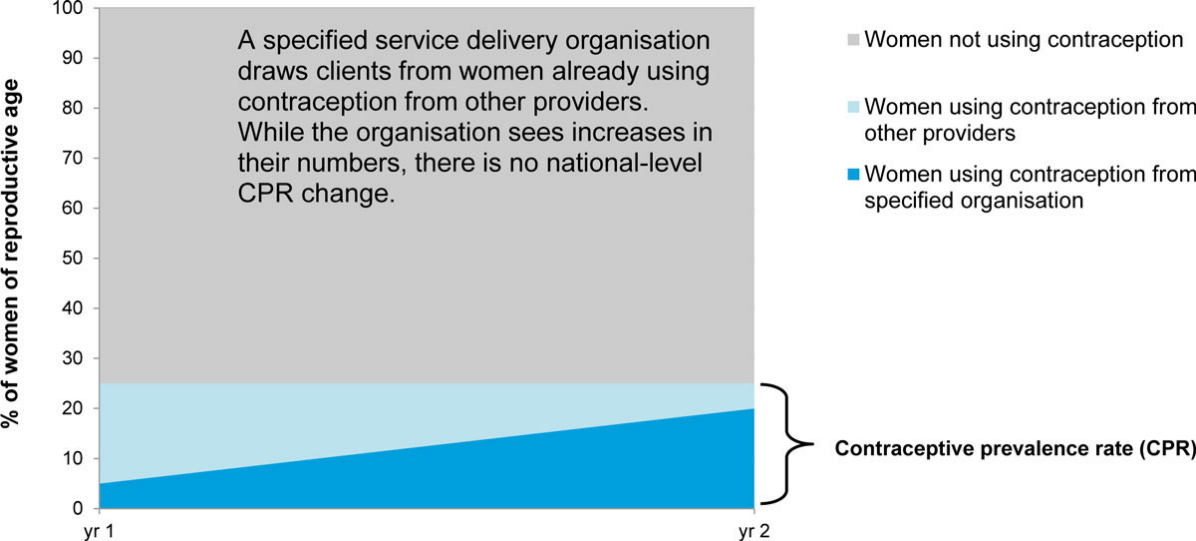
**Illustration of substitution effect on service provider's contribution to CPR**. In this example, an organisation draws additional users from women already using contraception from other sources (light blue). Therefore, even though the organisation saw an increase in its user numbers (growth in dark blue section), there was no net effect on the national CPR (light blue + dark blue), as it remained at 25%.

The main reason these metrics do not translate into national-level impact is the substitution of clients. Some clients who are new to a service delivery organisation are actually not new to family planning; rather, they previously received contraceptives from a different provider. Therefore, these clients' move to the new provider can increase an individual service delivery organisation's service and CYP numbers without having any effect on increasing CPR (Figure 1). A study in Honduras demonstrates this substitution effect, finding that increased sales of a new, socially-marketed oral contraceptive pill had no impact on national levels of contraceptive use; prior users were simply substituting their former contraceptives for the new brand [7].

Even if substitution could be addressed, using service outputs and CYPs to demonstrate an organisation's national impact presents another challenge. In order to determine any increase in modern CPR at the national level, the number of women using family planning in a given year needs to be estimated, a figure not indicated by annual service provision data and the resulting CYPs. This estimated number is more comprehensive than the number of family planning clients served in a given year because it takes LAPM clients from past years into account. Since LAPMs offer multiple years of coverage, women will continue to use these methods in years when they did not receive services. While formulas for CYPs acknowledge that LAPMs are used for multiple years, this metric sums together all of the future years of use for a method without defining the time period. For example, a programme generating 1,000 CYPs in 2012 could realise all of these in 2012 if they provide short-term methods alone, or the programme could produce the same number of CYPs over the next 10 years through the provision of certain LAPMs. Therefore, CYPs cannot be 'annualised' to produce an estimate of the number of women protected from pregnancy in a given year. To determine an organisation's impact on national CPR, contraceptive use within a year needs to be isolated, producing an estimate that includes the number of users served during that year along with the number of users continuing to use LAPMs received in previous years.

Several models already exist that examine the relationship between family planning services, CPR, and wider health outcomes. Most notably, the FamPlan module of the Spectrum modelling system [8] and the Realty √ [9] model demonstrate how projected changes in CPR result in different fertility rates, allowing estimations of changes in other health conditions, such as the number of unintended pregnancies, unsafe abortions, or maternal deaths. Unfortunately, while these models can be used to show the impact of CPR increases on reductions in national burdens of these conditions, they do not cater to the unique needs of service delivery organisations. These models cannot estimate an organisation's individual contribution to increasing CPR, which accounts for the substitution effect. Moreover, these existing models cannot transform service provision data into increasing CPR by modelling estimates of the number of family planning users in a given year. Therefore, the national health impact of an organisation's family planning programme cannot be determined.

**Figure 2:**
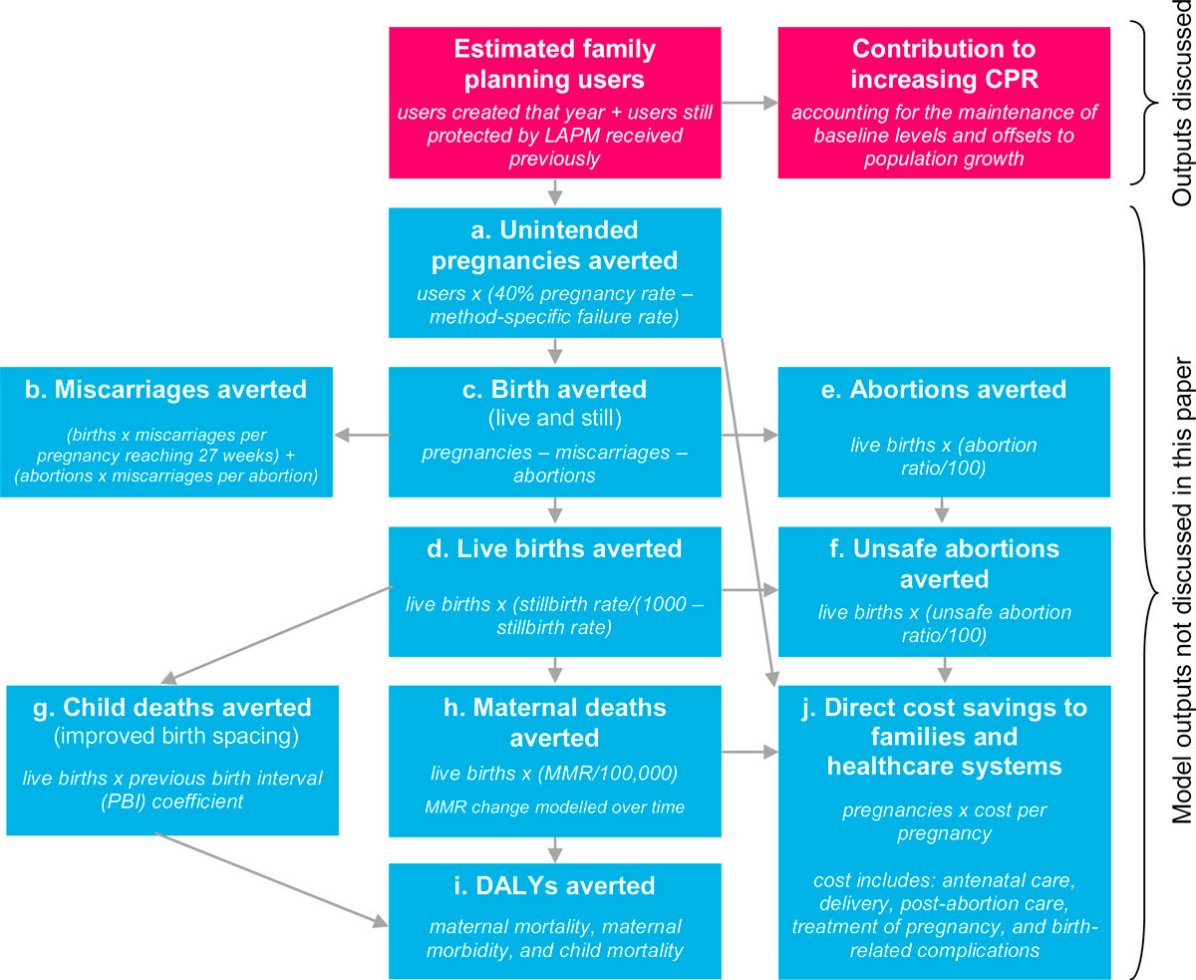
**Flowchart of model outputs estimated by Impact 2**. **a**. The 40% pregnancy rate is a comparison rate reflecting the chance of pregnancy had the women not been using contraception. **b**. Miscarriage estimates based on life tables of spontaneous abortion probabilities created by Hammerslough (1993) [16]. **c/d**. National stillbirth rates used are from Cousens et al. (2011) [17]. **e/f**. National abortion ratios used when published; otherwise, sub-regional ratios are used based on WHO and Guttmacher studies [12,18]. **g**. Methodology developed by Population Services International to estimate the incremental effect of birth spacing from large sub-regional demographic health survey datasets [19]. These estimates may be unreliable because data about the linkages between CPR, birth spacing, and child mortality are currently very limited, and will be improved as more research becomes available in 2013. **h**. Maternal mortality ratio (MMR) is modelled to change overtime based on several point estimates of MMR from WHO [13]. **i**. Disability-adjusted life years are calculated based on years of life lost (YLL) per each maternal and child death, and years of life lost to disability (YLD) from maternal conditions, estimated by applying a sub-regional ratio of YLD/YLL for maternal conditions [20]. **j**. Costs include supplies and direct labour only; default cost savings assume full coverage (i.e. all women who need care receive it), and draw on regional cost and incidence data from the Reproductive Health Costing Tool [21].

To fill these gaps, MSI has designed the Impact 2 model. This model is a tool that programme managers can use to easily and robustly estimate the impact of service provision, either past or future, on increasing national CPR. Impact 2 estimates the total number of family planning users in a given year, and what proportion of these users counts towards an organisation's contribution to increasing CPR among all women, or in-union women as specified by the user (fuchsia boxes, Figure 2 below). In doing so, the model considers the aforementioned challenges of how to account for the continuation of LAPM clients and the substitution effect. The Impact 2 model has wide uses beyond this function, enabling an organisation to estimate its impact using higher-level health, demographic, and economic metrics such as unintended pregnancies averted, maternal deaths averted, and disability-adjusted life years (DALYs) averted (blue boxes, Figure 2).

This paper describes the methodology behind the first level of the Impact 2 model, showing how service provision data can be translated into modelled user numbers and ultimately, the key model output at its top level: an organisation's estimated percentage point contribution to increasing the modern CPR. Following the model description, a demonstration of the model's outputs is presented, along with a discussion of the model's benefits and limitations. The methodology for calculating higher-level maternal and child health metrics offered by Impact 2 is not covered in this paper.

## Methods

### Model overview

Impact 2 is designed to convert an organisation's service delivery data, i.e. the number of services provided or commodities distributed to clients, into a range of results, including increasing CPR. First, these data are used to determine estimates of the number of women served by an organisation who are using a family planning method during a certain year. To derive these estimates, different modelling procedures are used for users of long-term and short-term methods due to different characteristics of the methods. Then, the model divides these estimated user numbers among those who will maintain the organisation's previous year's contribution to the CPR and those new users who will be counted towards the percentage point contribution to increasing CPR.

The Impact 2 model works at a micro level, an appropriate approach given it is designed for use by service delivery organisations. Impact 2 estimates the outcomes (e.g. unintended pregnancies averted) for an individual who is using family planning provided by a specific service delivery organisation. It attempts to isolate the impact of individual service delivery organisations, although it does not attempt to show indirect population-level changes, such as reductions in the total fertility rate (TFR) or the maternal mortality ratio (MMR).

Operating in Microsoft Excel, Impact 2 uses a user-friendly interface that leads the user through each step of the modelling process, from initial inputs to final outputs. The model is run on one country (or region) at a time and allows the user to look at any trend of the impact of family planning services from 2001 to 2020.

Note that the Impact 2 model produces results that show how an organisation was responsible for adding women to the national CPR. Because of the model's design, it does not consider if these women would have otherwise had access to family planning services from another provider. Thus, the results should be interpreted as what the organisation contributed to increasing CPR, but not how much lower the national CPR would have been if the programme never existed.

Experts at Guttmacher Institute, Futures Institute, Population Services International (PSI), International Planned Parenthood Foundation, and London School of Hygiene and Tropical Medicine have reviewed the Impact 2 model. It is freely available for non-commercial use at http://www.mariestopes.org/impact-2.

### Theoretical basis for dividing user estimates between baseline CPR levels and the contribution to increasing modern CPR

In order for an organisation to contribute to increasing the modern CPR, it cannot simply rely on counting services provided to women who were not already using contraception, called adopters. Increases are only realised on top of maintaining an organisation's baseline CPR contribution from the previous year. Once this baseline level is maintained, adopters can be counted towards increasing CPR.

There are two components involved in maintaining a baseline CPR contribution: 1) preserve the organisation's absolute number of users at baseline; and 2) reach additional users to offset population growth in those countries where populations are growing, a reality in most of the developing world. Note that population growth among women of reproductive age increases the absolute number of users needed each year to maintain an organisation's baseline CPR contribution because CPR is a proportional measure.

**Figure 3:**
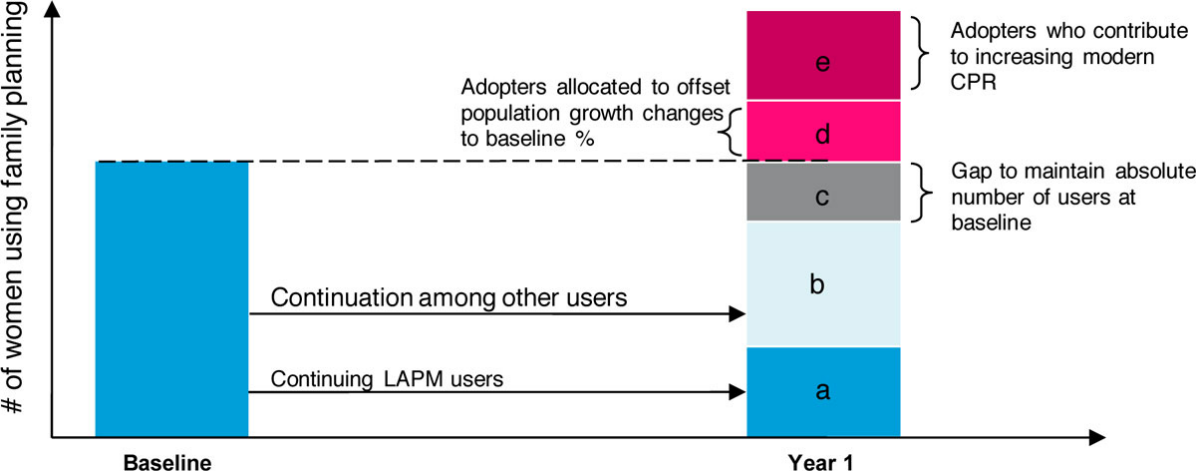
**Illustration of the allocation of an organisation's clients for determining its contribution towards increasing CPR**.

Women who are already using a contraceptive method from an organisation, and continue to use a method from the organisation, will count towards maintaining the organisation's baseline number of users. This principle also applies to women who decide to switch family planning methods, provided they continue to obtain services from the same organisation. Therefore, for each year of interest, an estimate of the number of women continuing to receive family planning services from the organisation is needed. For users of long-term methods, continuation is built into the model, so these estimates already exist (Figure 3, a). However, because the model is based on service provision data and not client-based data, it cannot account for:

• Short-term users receiving services from the organisation who continue to use contraception in future years (the model assumes that all discontinue at the end of the year); or

• LAPM users obtaining services from the organisation who receive another method after discontinuation (while service data will capture their resupply method, the data will not show their previous family planning method).

To address these shortcomings in the available data, Impact 2 uses an estimate of the proportion of clients that is continuing to receive services from the organisation as a proxy for continuation in these two groups (Figure 3, b).

In some cases, once all continuation has been accounted for, there can still be a gap between the number of continuing users and the number needed to maintain the previous year's number (Figure 3, c). This gap exists because some women may stop using family planning as they no longer have a need for contraception, or, they might stop visiting the service provider for other reasons. When this gap occurs, adopters must fill it.

Once it is ensured that the previous year's number of family planning users is maintained, a portion, and sometimes all, of the adopters needs to be allocated to maintaining the baseline CPR contribution in order to offset population growth, if necessary (Figure 3, d). Only adopters can be counted in this manner because the growth in users must be among women not already counted in the national CPR.

Finally, once all gaps in the baseline CPR level are filled, any remaining adopters can be counted as contributors towards increasing CPR (Figure 3, e). The model uses this proportion when determining the service provider's percentage point contribution to increasing modern CPR.

Note that Impact 2 cannot account for the family planning methods delivered by other service providers in a country because the model is designed for use by a single organisation. Therefore, in order for an organisation's estimated contribution to increasing CPR to translate into a national-level increase, an assumption must be made that all other providers maintain at least their baseline CPR contributions. If not, an organisation's estimated contribution to increasing CPR will help offset a decrease by another provider instead. In addition, women who change from another provider, called provider changers, do not contribute towards maintaining an organisation's baseline level or towards increasing CPR, because the CPR already included these women. This principle also applies to provider changers who switch methods, even more effective ones such as a switch from a short-term method to an LAPM. Impact 2 employs these principles in order to address the substitution of clients, ensuring that service delivery organisations are contributing to increasing CPR, not claiming a CPR contribution created by another organisation.

### Model inputs

Impact 2 determines an organisation's impact on increasing CPR and other health outcomes based on various inputs. Service delivery data are the primary input. Additional inputs include the organisation's client profile and a set of background data and assumptions regarding national demographics and maternal health outcomes, contraceptive methods used, and costs. As with any modelling exercise, the quality of the input data will dictate the quality of the results.

#### Service delivery data

The minimum data required to operate Impact 2 are an organisation's contraceptive service provision data by method and year. The user must enter this data for all years of interest. In addition, the model allows for historic service provision data (dating back to 1982) to be input so that an organisation can understand its impact over time, an important model feature given that some women who received services in the past may still be using the methods during the period of interest. These historic data are used to estimate the organisation's baseline contribution to increasing CPR, as discussed below. If these historic data are not entered into the model, the organisation will not be able to estimate an accurate baseline level.

#### Client profile

In order to account for substitution (i.e. existing modern contraception users who are new to the organisation), and effectively distribute users towards maintaining or increasing an organisation's CPR contribution, the model relies on a client profile that divides an organisation's clients from each year into three groups:

• % adopters: the proportion of clients who were not using a modern family planning method before receiving services (note: this includes women who have used modern contraception in the past (ever-use) but were not currently using before coming for services);

• % continuers: the proportion of clients who were already using a modern family planning method which they had received from the service delivery organisation; and

• % provider changers: the proportion of clients who were existing modern family planning users and who changed from a different provider to one affiliated with the service delivery organisation of interest.

The organisation inputs this client profile into the model, and can be based on client exit interviews or client registration forms, or estimated based on project design. More detailed guidance on how an organisation can estimate its client profile can be found in the Impact 2 training materials available on the MSI website.

#### Background data and assumptions

Impact 2 relies on a series of data and assumptions to model the organisation's service data into the various results. These data and assumptions include: national trends (e.g. population projections, fertility rate projections), CPR data, mortality data (MMR, age-specific mortality rates, unsafe abortion mortality), contraceptive method-specific assumptions (e.g. typical-use failure rates, median age of sterilisation), pregnancy outcomes (e.g. miscarriage rates, abortion ratios), and cost savings data (e.g. cost and incidence of pregnancy care and delivery). While any values can be entered into the model, default data and assumptions have been pre-loaded for all developing countries in order to improve the ease of use. These default values are based on Demographic and Health Surveys (DHS) [10], United Nations (UN) Population Prospects [11], World Health Organisation (WHO) studies [12,13], and numerous other validated sources (see Table 1 for sources of data referenced in this paper). MSI updates these default data and assumptions annually to ensure that Impact 2 models its outputs from the most current sources available.

**Table 1:** Sources of relevant default assumptions in Impact 2 model

Data	Source
Projected women of reproductive age	UN Population Prospects (2010) revision [11]

Projected female life expectancy at birth (e_0_)	UN Population Prospects (2010) revision [11]

Female age-specific mortality rates (_5_p_x_)	Model life table families [14]

Units needed for one year of coverage	United States Agency for International Development (USAID) 2011 CYP Update [6]

Median age of sterilisation	DHS (for samples greater than 30 women) or weighted regional average [10]

Cumulative continuation rates (IUD, implant)	USAID 2011 CYP Update [6]

### How the Impact 2 model operates

#### Determining estimates of users of long-term methods from an organisation

To estimate the number of clients using a long-acting or permanent method of family planning each year, Impact 2 creates virtual cohorts of users by method and year from an organisation's service provision data. Long-acting methods are implants and IUDs; permanent methods are male and female sterilisation. To estimate continued use in subsequent years, the model applies annual cumulative continuation rates (CCRs) (IUDs and implants) and mortality rates (sterilisation) to these cohorts. For users of IUDs and implants, the entire cohort is removed from the model when the maximum duration of use is reached. Among sterilisation users, removal occurs when the cohort is no longer of reproductive age, which is assumed to be 49 years. For male sterilisation, note that the female partner who is protected by the man's sterilisation is counted as the user because measures of contraceptive use are based on women using contraception.

For each long-acting method *m *(10-year IUD, 5-year IUD, 5-year implant, 4-year implant, 3-year implant), the number of users in the cohort created in year *y *is calculated for each year (*n *= 0 to *n *= maximum duration of the method):

Usersmy+n=ServicesprovidedmyAverageCCRn,CCRn+1

where *CCR_n _*is the cumulative continuation rate in year *n *for the specified method. Note that *CCR_0 _*= 1, since all women are using the method at the time of insertion.

For male and female sterilisation, the number of users in the cohort created in year *y *is calculated for each year (*n *= 0 to *n *= cohort reaches age 49), by applying the estimated probability of survival from one year into the next. Survival probabilities are not cumulative, meaning that the survival rate is applied to the previous year's user estimate, rather than the original number of male and female sterilisation clients in year *y*:

$${\text{Users}}^{\text{m}}{}_{\text{y+n}}{\text{= Users}}^{\text{m}}{}_{\text{y}}\times {\left({}_{5}{\text{p}}_{\text{x}}\right)}^{\frac{1}{5}}$$

where *_5_p_x _*is the probability of survival between ages *x *and *x+5 *for the age group that contains the median age of the cohort. The median age of the cohort is set to begin at the median age of sterilisation, which then increases by one year for each subsequent year. The probability of survival is raised to the one-fifth to reflect the probability of survival over one year rather than across the standard five-year interval for survival probabilities, assuming an even distribution of survival across the age group. The value of *_5_p_x _*is determined based on a country's assigned model life table family [14] and the projected life expectancy at birth (*e_x_*) in year *y+n*. When determining which model life table to use, MSI applies results from its own analyses to assign each country into one of nine model life table families: Coale-Demeny (CD) East, CD North, CD South, CD West, UN Chilean, UN Far East Asian, UN General, UN Latin, and UN South Asian.

**Figure 4:**
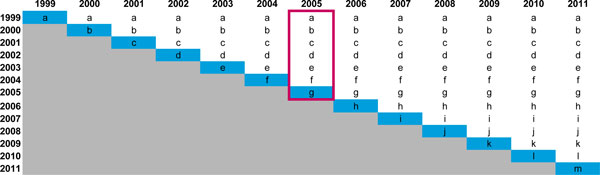
**Illustrative example of determining cohorts of LAPM users**. Impact 2 creates a matrix such as the one above for each LAPM used by a client of the organisation. Each row represents the cohort that corresponds to the year in which the method was delivered. For example, 'a' denotes the cohort provided contraceptive services in 1999. This cohort 'a' is shown in subsequent years to represent the users' continuation with their LAPM in future years. To estimate the total number of LAPM users of a long-acting and permanent method in any given year, the numbers associated with the column of the year of interest are totalled, as shown by the pink box surrounding the cohorts listed in 2005. In actuality, the model accounts for cohorts starting from services provided in 1982 to capture all continuation of LAPMs from 2001 (the first year of analysis included in the model) onwards.

Once the number of users for each cohort has been determined, Impact 2 calculates the estimated number of LAPM users in any given year by totalling the figures for each cohort (Figure 4).

#### Determining estimates of users of short-term methods from an organisation

Short-term family planning methods are those contraceptive methods that require multiple commodities for one year of coverage, such as oral contraceptive pills, condoms, and injectables. For these methods, virtual cohorts are not needed because services provided in one year do not provide protection beyond that year. Therefore, all short-term method users are counted in an organisation's annual service provision data for the subsequent years of use, even if a woman continues to use a short-term method from year to year.

The type of data collected by most service delivery organisations makes the estimation of short-term users difficult, however. In general, providers collect data on the number of commodities provided (e.g. number of contraceptive pill cycles) rather than the number of clients served. For instance, while an organisation's data might show the distribution of 13 pill cycles (the number needed for a full year of coverage), it is unknown if 13 different women each received one cycle of pills, if one woman received a full year's worth of pills, or if a different distribution was provided. Tracking client numbers requires a client-based management information system that can link multiple visits in a year to a single client, a system that is beyond the resources of most family planning providers in the developing world.

Despite these shortcomings with the data, it is possible to estimate the number of users of short-term methods. The ultimate aim is to derive an estimate of a method's users that is comparable to measures of CPR, which are determined by data from national population surveys, such as DHS. These surveys have their own limitations, however. DHS data capture contraceptive use at a single point in time over a given year, with 'current use' defined by the respondent [15]. Depending on when the survey is undertaken and how the potential pill clients (anywhere from 1 to 13 clients) are distributed across the year (i.e. consecutively versus concurrently), it is possible for a survey to detect all or none of them.

For this reason, the Impact 2 model takes a conservative approach, counting 'users' as having full coverage for the entire year. Thus, in the preceding example, the 13 pill cycles are attributed to a single contraceptive pill user over the course of the year. Assuming even distribution of use across the year, the estimated number of pill users captured by the organisation's service delivery data should accurately reflect the user numbers captured in the CPR. If use were actually skewed (e.g. multiple clients who simultaneously take one cycle of pills each), the user estimates may not accurately reflect the same pill use captured by the CPR.

Therefore, Impact 2 estimates the number of short-term method users for each method *m *as follows:

usersmy=ServicesprovidedmyUnitsneededfor1yearofcoveragem

The number of units needed for one year of contraceptive coverage represents the number of commodities needed. This number is similar to the one used to calculate couple-years of protection (CYP) [6], but it is not identical because method failure rates are not included. Impact 2 accounts for method failure when estimating unintended pregnancies to users. It should be noted that Impact 2 assumes that short-term user estimates refer to clients who use the short-term contraceptive methods according to the general usage patterns applied by USAID when calculating CYP factors [6]. Inconsistent use that may lead to method failure is accounted for elsewhere in the model, when method-specific failure rates are applied to estimate unintended pregnancies averted (not covered in this paper).

#### Determining an organisation's contribution to increasing modern CPR

This section describes each of the calculations undertaken by Impact 2 to estimate an organisation's contribution towards increasing modern CPR. It is explained in a series of steps.

##### 1. Establish a baseline CPR contribution from the previous year

An organisation's baseline contribution to CPR is calculated in terms of its absolute contribution (i.e. baseline user numbers) and relative contribution (i.e. baseline CPR contribution which accounts for population growth offsets). Both are based on its contributions in the baseline year (*y_0_*), which is the year before the selected trend starts. For example, a trend from 2005 to 2010 is measured against a 2004 baseline.

The number of baseline users counts all users in the baseline year, using the methodology described above for modelling service provision data into estimates of LAPM and short-term method users. The baseline estimate is made from historic service provision data, which the service delivery organisation must input into the model:

$$\text{Baseline}\;\text{users = Total}\;\text{number}\;\text{of}\;\text{user}{\text{s}}_{{\text{y}}_{\text{0}}}$$

To estimate the baseline CPR contribution, divide the total estimated number of users in the baseline year by the projected number of women of reproductive age in the same year. If the survey-based CPR estimates entered into the model are based on all women, then, the full WRA number is used; otherwise, if the CPR estimates are for married or cohabitating women (hereafter referred to as 'in-union'), then the WRA is multiplied by the estimated proportion of women in union:

$$\text{Baseline}\;\text{CPR}\;\text{contribution}=\;\frac{\text{Total}\;\text{number}\;\text{of}\;{\text{users}}_{{\text{y}}_{\text{0}}}}{\text{Number}\;\text{of}{\left(\text{WRA}\;\text{or}\;\text{in-union}\;\text{WRA}\right)}_{{\text{y}}_{\text{0}}}}$$

##### 2. Account for pre-existing LAPM users

Pre-existing users are the estimated number of LAPM users each year that had received LAPM services before the year of interest (i.e. baseline year and before). These numbers are calculated by method and year from the virtual cohorts of LAPM users created in the baseline year as well as in the years prior to baseline for which service provision data were entered. The total number of women estimated to still be using the long-term method during each year of the trend (*y_1 _*to *y_n_*) is summed across each cohort (see Figure 4 above).

##### 3. Calculate the number of new users created each year from all family planning methods

The service provision data entered for the years covered by the trend (*y_1 _*to *y_n_*) are converted into the estimated number of new users created the year the services are provided, for both LAPMs and short-term methods. To calculate this number, Impact 2 follows a similar methodology to that described above for determining estimates of short-term and long-term users. However, the model only calculates estimates of LAPM users for the first year. Note that the number of first-year LAPM users will be slightly fewer than the number of clients served in the first year because some women may die, and some women may discontinue the method before the end of the first year. Below are the formulas that Impact 2 uses for each type of contraceptive method:

• For each long-acting method *m *(10-year IUD, 5-year IUD, 5-year implant, 4-year implant, 3-year implant), the number of users created in year *y *is calculated as follows:

Newusersmy1=Servicesprovidedmy1Average(CCR0,CCR1)

where *CCRn *is the cumulative continuation rate in year *n *for the specified long-term method. Note that *CCR_0 _*= 1, since all women are using the method at the time of insertion.

• For male and female sterilisation, the number of users created in year *y *is calculated as follows:

$${{\text{New users}}^{\text{m}}}_{{\text{y}}_{1}}={{\text{Services provided}}^{\text{m}}}_{{\text{y}}_{\text{1}}}\times {\left({}_{5}{\text{p}}_{\text{x}}\right)}^{\frac{1}{5}}$$

where *_5_p_x _*is the probability of survival between ages *x *and *x+5 *for the age group that contains the median age of sterilisation. See above for the source for calculating *_5_p_x_*.

• For each of the short-term methods, user numbers are calculated by dividing the number of services provided by the number of units needed for one year of contraceptive coverage:

Newusersmy1=Servicesprovidedmy1Unitsneededfor1yearofcoveragem

##### 4. Distribute first-year users between maintaining baseline and increasing user numbers

First, Impact 2 makes an estimate of the total number of users needed to maintain the baseline number of users:

$$\text{Users}\;\text{needed}\;\text{to}\;\text{maintai}{\text{n}}_{{\text{y}}_{1}}\text{= Baseline}\;\text{users - Pre-existing}\;\text{user}{\text{s}}_{{\text{y}}_{1}}$$

Then, the model checks to see how the new users should be distributed, based on the numbers needed and the client profile. To do so, the model initially calculates the total number of new users who will be apportioned as continuers and adopters:

$$\text{New}\;\text{continuer}{\text{s}}_{{\text{y}}_{\text{1}}}=\text{New}\;\text{user}{\text{s}}_{{\text{y}}_{\text{1}}}\times \text{\% continuer}{\text{s}}_{{\text{y}}_{\text{1}}}$$

$$\text{New}\;\text{adopter}{\text{s}}_{{\text{y}}_{\text{1}}}=\text{New}\;\text{user}{\text{s}}_{{\text{y}}_{\text{1}}}\times \text{\% adopter}{\text{s}}_{{\text{y}}_{\text{1}}}$$

There are three possible situations in this apportionment:

Situation 1: There are enough new continuers to maintain the baseline contribution, and therefore, all adopters are allocated to increasing user numbers;

Situation 2: There are not enough new continuers or adopters to maintain the baseline contribution, and therefore, all continuers and adopters are allocated to maintaining baseline levels, with the number of users actually decreasing from the baseline contribution; and

Situation 3: There are not enough new continuers to maintain the baseline number, and therefore, some adopters are allocated to maintaining the baseline contribution, with the remaining adopters allotted to increasing user numbers.

The model checks to see which situation is occurring, then distributes new users accordingly, into two groups:

$$\text{Maintain}\;\text{user}{\text{s}}_{{\text{y}}_{\text{1}}}=\text{New}\;\text{users}\;\text{that}\;\text{maintain}\;\text{the}\;\text{baseline}\;\text{user}\;\text{numbe}{\text{r}}_{{\text{y}}_{\text{1}}}$$

$$\text{Increase}\;\text{user}{\text{s}}_{{\text{y}}_{\text{1}}}=\text{New}\;\text{users}\;\text{that}\;\text{increase}\;\text{numbers}\;\text{above}\;\text{the}\;\text{baseline}\;\text{leve}{\text{l}}_{{\text{y}}_{\text{1}}}$$

For situation 1, all adopters are allotted to increasing user numbers, therefore:

$$\text{Maintain}\;\text{user}{\text{s}}_{{\text{y}}_{\text{1}}}=\text{Baseline}\;\text{users - Pre-existing}\;\text{user}{\text{s}}_{{\text{y}}_{\text{1}}}$$

$$\text{Increase}\;\text{user}{\text{s}}_{{\text{y}}_{\text{1}}}=\text{New}\;\text{adopter}{\text{s}}_{{\text{y}}_{\text{1}}}$$

For situation 2, all adopters are allocated to maintaining the baseline user number, resulting in:

$$\text{Maintain}\;\text{user}{\text{s}}_{{\text{y}}_{\text{1}}}=\text{New}\;\text{adopter}{\text{s}}_{{\text{y}}_{\text{1}}}\text{+ New}\;\text{continuer}{\text{s}}_{{\text{y}}_{\text{1}}}$$

$$\text{Increase}\;\text{user}{\text{s}}_{{\text{y}}_{\text{1}}}=\text{0}\;$$

For situation 3, some adopters will be allocated to both maintaining the baseline user number and increasing user numbers above baseline, so:

$$\text{Maintain}\;\text{user}{\text{s}}_{{\text{y}}_{\text{1}}}=\text{Baseline}\;\text{users - Pre-existing}\;\text{user}{\text{s}}_{{\text{y}}_{\text{1}}}$$

$$\text{Increase}\;\text{user}{\text{s}}_{{\text{y}}_{\text{1}}}=\text{New}\;\text{adopter}{\text{s}}_{{\text{y}}_{\text{1}}}\text{- }\left[\text{Baseline}\;\text{users - Pre-existing}\;\text{user}{\text{s}}_{{\text{y}}_{\text{1}}}\text{- New}\;\text{continuer}{\text{s}}_{{\text{y}}_{\text{1}}}\right]$$

##### 5. Calculate the proportion of new users counting towards CPR in the first year

Now, Impact 2 estimates the proportion of all new users counting towards CPR in the first year. To do so, the number of users maintaining the baseline contribution and the number of users above the baseline level are divided by the total number of new users created in the first year. Therefore:

$$\text{\% counting}\;\text{in}\;\text{CP}{\text{R}}_{{\text{y}}_{\text{1}}}=\;\frac{\text{Maintain}\;\text{user}{\text{s}}_{{\text{y}}_{\text{1}}}\text{+ Increase}\;\text{user}{\text{s}}_{{\text{y}}_{\text{1}\;}}}{\text{New}\;\text{user}{\text{s}}_{{\text{y}}_{\text{1}}}}$$

This proportion is applied to the number of new users of each method *m *to estimate the number of users of each method that contributes to a CPR increase:

$$\text{New}\;\text{CPR}\;\text{user}{{\text{s}}^{\text{m}}}_{{\text{y}}_{\text{1}}}=\text{\% counting}\;\text{in}\;\text{CP}{\text{R}}_{{\text{y}}_{\text{1}}}\times \;\text{New}\;\text{user}{{\text{s}}^{\text{m}}}_{{\text{y}}_{\text{1}}}$$

##### 6. Calculate the number of LAPM users who will continue the method in subsequent years

Once the number of new users who count towards CPR is determined, an additional step must be taken for the new LAPM users counting in the CPR. For these users, an estimate is needed of how many will still continue to use their LAPM in each subsequent year left in the trend. These users are referred to as 'existing users', which are different from pre-existing users (who were provided services *before *the first year of the trend). To determine the number of these existing users, Impact 2 follows the methodology described earlier for calculating estimates of long-term users. Thus, for each LAPM method *m*, the virtual cohort of users will be followed, applying the corresponding continuation rates (based on year) and mortality rates (based on age of cohort and life expectancy) each year.

Note that there are not any existing users in the first year (*y_1_*). All clients who receive services are counted as new users, and any past clients who continue to use their LAPM are counted as pre-existing users.

##### 7. Repeat this procedure (steps 1-6) for the remaining years in the trend

Impact 2 repeats this process for each remaining year in the trend. However, rather than measuring against the baseline year, calculations are based on the year immediately prior to the year of interest. For example, the second year in the trend (*y_2_*) measures increases against the total number of users contributing to CPR in the first year (*y_1_*).

The total number of users contributing to a CPR increase in a given year (*y_z_*) is calculated as follows:

$$\text{Total}\;\text{users}\;\text{in}\;{\text{CPR}}_{{\text{y}}_{\text{z}}}=\text{Pre-existing}\;\text{user}{\text{s}}_{{\text{y}}_{\text{z}}}\text{+ Existing}\;\text{user}{\text{s}}_{{\text{y}}_{\text{z}}}\text{+ Maintain}\;\text{user}{\text{s}}_{{\text{y}}_{\text{z}}}\text{+ Increase}\;\text{user}{\text{s}}_{{\text{y}}_{\text{z}}}$$

Once the total number of users in the CPR in year *y_z _*is known, the number of users needed to maintain this number of total users in the CPR in the following year (*y_z+1_*) can be calculated using the following:

$$\text{Users}\;\text{needed}\;\text{to}\;\text{maintai}{\text{n}}_{{\text{y}}_{\text{z+1}}}=\text{Total}\;\text{users}\;\text{in}\;\text{CP}{\text{R}}_{{\text{y}}_{\text{z}}}\text{- Pre-existing}\;\text{user}{\text{s}}_{{\text{y}}_{\text{z+1}}}\text{- Existing}\;\text{user}{\text{s}}_{{\text{y}}_{\text{z+1}}}$$

As described above in step 4, allocation of new users is based on the three scenarios for continuers and adopters. Thus, new users are distributed accordingly into:

$$\text{Maintain}\;\text{user}{\text{s}}_{{\text{y}}_{\text{z+1}}}=\text{New}\;\text{users}\;\text{to}\;\text{maintain}\;\text{the}\;\text{previous}\;\text{year's}\;\text{number}\;$$

$$\text{Increase}\;\text{user}{\text{s}}_{{\text{y}}_{\text{z+1}}}=\text{New}\;\text{users}\;\text{that}\;\text{increase}\;\text{the}\;\text{previous}\;\text{year's}\;\text{number}\;$$

Next, as described above in step 5, the percentage of new users, of both long-term and short-term methods, that contributes to an organisation's CPR increase is estimated. As noted in step 6, the proportion of LAPM users must be input into virtual cohorts, which are traced out to estimate the number of existing users in each of the trend's remaining years. This iterative process is continued for each successive year, until the end of the trend is reached.

##### 8. Produce the model output: the percentage point contribution to increasing CPR

The final Impact 2 output described in this paper is an organisation's estimated percentage point contribution to increasing modern CPR. In other words, this figure is an estimate of how much a service delivery organisation has, or will, increase the national CPR from its baseline contribution, assuming all other providers at least maintain their baseline contributions.

To estimate this figure, an organisation's total percentage point contribution to increasing CPR is calculated for each year first. To do so, Impact 2 divides the total number of users counting in CPR each year (total users in CPR in year *y_z_*) by the number of WRA in each year (or in-union WRA if the CPR of only in-union WRA is selected):

$$\text{Total}\;\text{\% point}\;{\text{contribution}}_{{\text{y}}_{\text{z}}}=\;\frac{\text{Total}\;\text{users}\;\text{in}\;{\text{CPR}}_{{\text{y}}_{\text{z}\;}}}{\text{Number}\;\text{of }{\left(\text{WRA}\;\text{or}\;\text{in-union}\;\text{WRA}\right)}_{{\text{y}}_{\text{z}}}}$$

This calculation's result includes the organisation's baseline percentage point contribution to CPR (since those users who maintain the baseline level are included in the total number of users counting in CPR). Therefore, to isolate the proportion contributing only to increasing CPR, the baseline percentage point contribution must be subtracted:

$$\text{\% point}\;\text{contribution}\;\text{to}\;\text{increasing}\;\text{CP}{\text{R}}_{{\text{y}}_{\text{z}}}=\text{Total}\;\text{\% }\text{point}\;\text{contributio}{\text{n}}_{{\text{y}}_{\text{z}}}\text{- Baseline}\;\text{CPR}\;\text{contributio}{\text{n}}_{{\text{y}}_{\text{0}}}$$

## Results

Actual results from Impact 2 are instructive for demonstrating how the model operates. Service provision data and a client profile from MSI's field office in Madagascar, Marie Stopes Madagascar, were fed into Impact 2 to estimate user numbers and determine the organisation's contribution to increasing modern CPR. Marie Stopes Madagascar has been providing reproductive health services in this sub-Saharan African country since the mid-1990s. In 2011, Marie Stopes Madagascar delivered services through 15 clinics, 12 outreach teams, and 133 social franchise outlets.

Additional file 1 shows Marie Stopes Madagascar's service provision data from 1996-2010. In addition, the following client profile was used for all years, based on unpublished data from client exit interviews that MSI conducted with 438 randomly selected clients in 2011:

• % adopters = 22.4%

• % continuers = 49.2%

• % provider changers = 28.4%

Using the data in Additional file 1 as well as the default assumptions preloaded into the model for Madagascar, Impact 2 calculated the trends of user numbers and CPR increases from 2005 to 2011. Condom users were excluded from the calculations to avoid the risk of overestimating condom use because of user wastage and dual protection (using condoms and another contraceptive method). MSI generally recommends that researchers and programmers calculate contributions to increasing CPR without condom use included.

In 2011, an estimated 200,000 women were using a contraceptive method provided by Marie Stopes Madagascar, with nearly 90% of these women using a LAPM (Table 2). From 2005-2011, Marie Stopes Madagascar steadily increased its family planning service provision, with the estimated number of all users increasing eightfold. The number of new adopters contributing to increasing CPR rose accordingly, also increasing eight times within this same time period. The number of adopters increasing CPR was slightly smaller than the total number of adopters served during each year of interest because some adopters are allocated to maintaining the previous year's (or baseline) CPR contribution. Table 2 shows a complete breakdown of the model estimates for different categories of users by year.

**Table 2:** Estimated number of women using family planning services from Marie Stopes Madagascar, 2005-2011, by type of user (excluding condoms)

	2005	2006	2007	2008	2009	2010	2011
Total users	26,244	33,525	46,255	55,550	83,317	141,038	197,860

LAPM users	14,105	21,371	31,636	44,109	73,427	123,768	175,283

Short-term method users	12,139	12,154	14,619	11,442	9,890	17,270	22,577

Users served each year	17,617	20,387	26,273	25,708	41,861	73,315	85,573

Adopters served each year	3,945	4,566	5,884	5,757	9,375	16,419	19,164

Adopters increasing CPR each year	2,489	3,842	5,005	4,676	8,085	14,793	17,005

Following the methodology described above, Impact 2 calculated the 2005-2011 baseline CPR and increasing CPR contributions from these estimated user numbers. The baseline CPR contribution was estimated to be 0.5% points; in other words, in 2004, Marie Stopes Madagascar provided contraceptive methods to 0.5% of women of reproductive age. Beyond maintaining this baseline CPR contribution, Marie Stopes Madagascar is estimated to have contributed 1.2% points towards increasing the national modern CPR from 2005-2011. Due to the design of the model, this result can only be interpreted as an increase if the assumption that all other providers at least maintained their baseline (i.e. 2004) CPR contributions is true.

**Figure 5:**
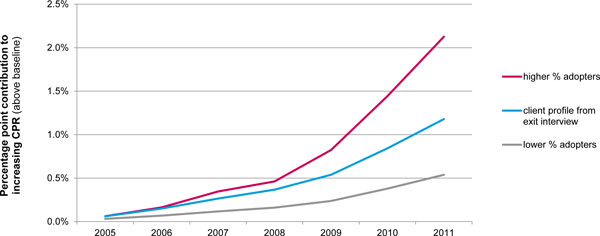
**Range of Marie Stopes Madagascar's estimated contribution to increasing CPR, 2005-2011, based on different client profiles (excluding condoms)**. Marie Stopes Madagascar's estimated percentage point contribution to increasing modern CPR from 2005-2011 changes, depending upon the client profile input into the Impact 2 model. The middle line (blue) shows the model outputs based on the actual profile determined by 2011 exit interviews: 22% adopters, 49% continuers, and 28% provider changers. The bottom line (grey) displays results based on a lower percentage of adopters in the profile: 11% adopters, 61% continuers, and 28% provider changers. The top line (fuschia) shows the model estimates based on a higher percentage of adopters in the profile: 44% adopters, 28% continuers, and 28% provider changers.

To show the important role of the client profile in estimating CPR increases, the percentage point increase to modern CPR was recalculated based on two different client profiles: one with a lower percentage of adopters and one with a higher percentage of adopters than the actual client profile determined from exit interviews conducted in 2011. Running these different scenarios in Impact 2 is important when data for each year of interest are unavailable. In this case, Marie Stopes Madagascar only had exit interview data from 2011, so the client profile from this year needed to serve as a proxy for the other years in the trend (2005-2010). As it is unlikely that the 2011 client profile accurately applied to these years, it is useful to input different client profiles to model a range of possible results. As shown in Figure 5, the client profile with the highest proportion of adopters yielded considerably higher estimates of the percentage point contribution to increasing CPR.

**Figure 6:**
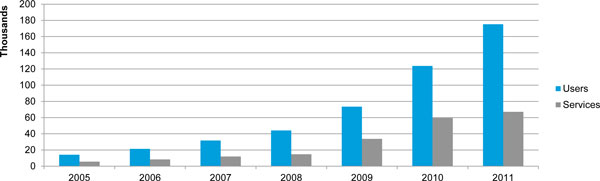
**Comparison of modelled LAPM user numbers with LAPM services provided each year, Marie Stopes Madagascar, 2005-2011**. The number of estimated LAPM users served by Marie Stopes Madagascar is considerably larger than the number of LAPM services tracked in its service provision data during a given year. The blue bars represent the number of women that Impact 2 modelled as using a LAPM method from Marie Stopes Madagascar in each year. Included in these estimates are pre-existing users of LAPMs who are modelled to still be using the method in the year of interest. The grey bars show the number of LAPM services provided each year to Marie Stopes Madagascar clients. These service provision data do not recognize continuation among users, something Impact 2 takes into account.

To illustrate the importance of accounting for continuation among LAPM users from year to year and not merely relying on service provision data, Figure 6 shows the difference between the number of women served with long-term methods and the estimated LAPM user numbers modelled by Impact 2. The modelled numbers are more than double those tracked by Marie Stopes Madagascar's service provision data because Impact 2 accounts for continuers when determining its estimates for users of long-term methods.

## Discussion

The Impact 2 model offers some important advantages for modelling a service delivery organisation's health impact in a country as the example from Marie Stopes Madagascar demonstrates. Namely, these results show that Impact 2's outputs can provide a service delivery organisation with a better understanding of its individual contribution to national CPR. Instead of relying on service provision data or CYPs that can misrepresent programme impact, an organisation can use Impact 2 estimates to gain a more accurate picture of the results of its efforts. These estimates also provide a more nuanced understanding of how whom an organisation reaches can greatly influence outcomes.

As shown, estimates of increasing CPR generated by Impact 2 isolate the contribution of an individual service delivery organisation, a process that other family planning models do not currently offer. With Impact 2, an organisation can better attribute CPR changes to its specific programme efforts. Moreover, because the analyses can look retrospectively, any CPR increase can be benchmarked against actual DHS data, obviating the need for the assumption that all other providers at least maintained their baseline CPR contributions. Through these comparisons, an organisation can interpret its programme impact on increasing CPR with reasonable certainty, given the organisation is confident in its client profile data. For example, in the Madagascar case described above, DHS data show modern CPR increasing from 14% in 2004 to 23% in 2009 [10]. Thus, assuming the client profile is accurate, Impact 2 results indicate that Marie Stopes Madagascar's service provision contributed around 4% of the growth in the CPR.

Besides isolating a specific organisation's impact, modelling user numbers with Impact 2 results in richer data about programme reach and more robust information about programme impacts than measures in current use, such as standard service provision data and CYPs. As shown in Figure 6, the overall number of LAPM users increases at a much faster rate than scale up of service provision due to the continuation of LAPM users from year to year. In 2011, for example, Marie Stopes Madagascar recorded the delivery of 67,111 LAPM services (Additional file 1), a much smaller figure than the 175,283 LAPM users that Impact 2 estimated for that year (Table 2). Thus, if an organisation relies solely on annual service provision totals for understanding programme impact, it can underestimate the number of users it serves, resulting in lower impact estimates. While CYPs are able to account for differences in usage duration of a contraceptive method, the total number of CYPs delivered in a year by a service organisation cannot be compared to annual impact measures such as increasing CPR. The reason why this comparison cannot be made is because CYPs are realised over an undefined number of future years, and therefore, this metric cannot be determined on an annual basis. Impact 2 has the added advantage of creating annual figures that holistically capture the full impact of an organisation's service provision (e.g. number of pregnancies averted, number of family planning users).

An organisation can also overstate its contribution to national-level changes if it simply looks at its total estimated user numbers, without accounting for substitution, as well as the dynamics of population growth and CPR changes. Impact 2 needed to discount a proportion of Marie Stopes Madagascar's estimated users from 2005-2011 to account for substitution, and allocate the majority of the remaining users to maintaining the baseline CPR contribution. In each of these years, only about 10% of the organisation's estimated users were considered as contributors to increasing CPR. If the total increase in users from 2005 to 2011, nearly 200,000 women, was attributed to increasing CPR, Marie Stopes Madagascar would have grossly overstated its program's contribution to increasing CPR. Using Impact 2 helps curb any unrealistic claims of impact.

The results above also underscore the importance of reaching adopters if an organisation wishes to have a measurable impact on increasing CPR. As explained, only adopters are able to contribute to increasing CPR. Figure 5 demonstrates how increases in the proportion of adopters in the client profile can dramatically affect the estimated percentage point contribution to increasing modern CPR. For example, if Marie Stopes Madagascar reached twice as many adopters, contribution to increasing CPR would be nearly doubled. However, these results also demonstrate that reaching adopters alone will not translate into CPR increases. As shown in Table 2, not all adopters served by Marie Stopes Madagascar contribute to increasing CPR. Impact 2 must account for maintaining a proportional contribution (i.e. percentage of WRA using a method from the provider), meaning some, if not all, adopters must go towards offsetting population growth. Moreover, the model recognises that some existing users will discontinue and need to be replaced by new adopters. Approximately 14% of the adopters from 2005-2011 had to be allocated towards maintaining a baseline contribution from the previous year. Therefore, it is essential for service delivery organisations to concentrate programming efforts on continuing to provide services to existing users as well as reaching adopters, to ensure that the maximum number of adopters is counted towards increasing modern CPR. The importance of maintaining this existing contribution should not be underestimated, especially in rapidly growing countries where much scale up is needed just to keep up with population growth.

### Limitations

While Impact 2 has made large improvements in how service delivery organisations are able to consider their health impact, they are still only modelled estimates, which are inevitably imprecise. Where applicable, the most conservative approach available was taken to minimize overstating results. A summary of key limitations is presented in Table 3, with additional discussion in the subsequent paragraphs. It is useful to separate limitations due to the quality of the input data from those inherent to the model design.

**Table 3:** Summary of key limitations related to data inputs and model methodology

Area	Limitation	Ability to minimize
**Data-related limitations **		

Service provision data	Service data must reflect services provided directly to clients; potential challenges include ensuring data quality, and inflation when counting commodities further back in supply chain.	Model is best positioned to be used by organisations delivering services directly to clients. Organisations should ensure their data accurately reflect services provided.

Client profile data	High potential to over- or underestimate CPR contribution depending on accuracy of client profile data.	High and low estimates created based on alternative client profiles.

National assumptions (e.g. mortality rates, discontinuation rates, population projections)	Limited potential to over- or underestimate results.	Best available data have been pre-loaded, with potential error minimised as much as possible.

**Model methodology-related limitations**		

Converting services to short-term users	Potential to either over- or underestimate number of users due to uncertainty around how many of the delivered short-term commodities are actually used. For coitus-based methods, there are limited data on units needed in a year, and the potential that dual protection double counts users.	Remove condom users from calculations to reduce greatest uncertainty. For pills and injectables, the approach minimises overestimation as much as possible given current data on consistency of use.

Treatment of provider changers	Potential to underestimate increases in CPR as model assumes provider changers are not replaced, and that women leaving the organisation (e.g. women who stop using contraceptives) are not counted in the CPR.	High and low estimates created based on alternative client profiles.

As an organisation's service provision data are the primary driver of Impact 2 results, inputting accurate data is critical for avoiding the inflation of user estimates. Therefore, the service data entered into the model must reflect only the services and commodities that reach clients. To do so, data collection systems need to be built with mechanisms that ensure the quality and accuracy of their data, an important underlying principle of all service data collection, not just for Impact 2 modelling. In cases where an organisation only operates in supply chain stages before the direct provision to clients (e.g. procuring contraceptives or selling contraceptives to pharmacies), a discount factor may need to be added to account for commodity wastage. In this situation, attribution must also be considered to avoid double counting.

The client profile presents another key data input challenge for the 'increasing CPR' metric as the methodology for estimating CPR increases is very dependent on this profile. As noted earlier, if data are not available for all years of interest, one year's profile is used as a proxy for the other years. Doing so is likely to produce inaccurate results because client profiles are expected to change from year to year. In addition, how client profile data are collected is likely to influence results (e.g. exit interviews that ask about contraceptive use in the past three months, versus a routine data system). Guidance on developing client profiles is available on MSI's website. These caveats underscore the importance of re-running the model with different client profiles to understand how its outputs change, as shown above in Figure 5.

Another potential shortfall is the model's treatment of provider changers, women who had used a family planning method previously and changed service providers. When estimating CPR increases, these women are excluded because the model sees changing providers as taking market share from another provider rather than growing the market. In reality however, there may be a complex exchange between providers. Because the model only accounts for one organisation, it is unable to determine if provider changers were replaced by their previous provider, or if clients who left the organisation went elsewhere or simply stopped using contraceptives. The model assumes the most conservative scenario in that all 'provider changers' would have continued to receive family planning services anyway and that all clients who leave the organisation have stopped using contraceptives. This assumption means that the model is likely to underestimate an organisation's contribution to increasing CPR. There are situations where these assumptions do not hold and it may require a revision of the client profile used in the model. For example, if the previous provider has shut down, these women may have stopped using contraceptives (and therefore, dropped out of the CPR) had they not changed providers. In this case, the organisation may wish to count these clients towards maintaining a CPR contribution, rather than excluding them altogether. To do so, the organisation can edit the client profile, with a proportion of the provider changers added to the proportion of continuers.

Impact 2's reliance on demographic projections from the United Nations on fertility rates (e.g. Total Fertility Rate) and demographics (e.g. WRA) may also be viewed by some as a limitation. Due to the use of these data, the model is unable to account for a dynamic relationship between increased contraceptive use, fertility rates, population growth, and age structure. However, because Impact 2 works on a relatively short time frame, the projected WRA population used to estimate CPR contributions will not be affected by short-term changes in the CPR and TFR, due to a lag between the emergence of smaller birth cohorts and the time when these cohorts reach reproductive age. Therefore, the micro level results from this model are still useful and relevant.

One important limitation of the increasing CPR metric itself is that because a woman is counted in the modern CPR no matter what contraceptive method she uses, the metric does not reflect any added benefit of existing users switching to more effective methods. Furthermore, in some cases, these switchers are fully excluded from the increasing CPR calculation, despite making this important change in method, because of the substitution effect. For example, a method switcher who changes providers in order to acquire the new method is not counted as part of the organisation's contribution to increasing CPR. While not captured within the increasing CPR metric, Impact 2 is capable of modelling the differential health impacts that result from the use of more effective family planning methods. Other Impact 2 model outputs, such as unintended pregnancies averted, factor in method switchers who opt for more effective methods. Future publications on the Impact 2 model will discuss these benefits.

Finally, many other benefits of providing family planning services are challenging, if not impossible, for Impact 2 to quantify. For example, the model cannot easily assess the role that service quality plays in discontinuation rates and method failure rates. Quantitative data also cannot describe how improved birth spacing enhances a mother's and a family's well-being, or what structural barriers exist in the country to prevent women from accessing the contraceptive services they need. Therefore, results from Impact 2 should always be placed within the national context, and supplemented with additional data from qualitative studies and other sources.

## Conclusion

With Marie Stopes International's Impact 2 model, service delivery organisations are better equipped to plan for and demonstrate their progress towards key global health goals, such as reducing the unmet need for family planning. By considering health impact from the perspective of an individual service delivery organisation, Impact 2 fills the gap not met by other models for family planning service outcomes. With this model, an organisation's service provision data can be converted into more useful measures of impact than simply counting the number of services and commodities provided, the number of clients served, or the number of couple-years of protection afforded. It does so while accounting for complex issues germane to family planning service delivery and essential to consider when developing effective metrics to measure impact:

• First, maintaining the existing CPR cannot be overlooked when estimating a service delivery organisation's contribution to increasing national CPR. Metrics that only look at adopters reached, without accounting for the nuances of population growth and maintenance of previous usage levels, will not fully capture the impact of services on changing CPR.

• Second, the model stresses the importance of accounting for substitution, i.e. providing services to women who were already using family planning services from another source. While a scale up in service delivery numbers will translate to a scale up in total impacts, this increase may not transfer into larger national contributions, however. Metrics that only capture scale (such as service provision numbers or CYPs) are unable to address substitution.

• Last, the model incorporates the issue of continuation among LAPM users into an annual output measurement. Metrics that only consider clients served within the raw data's year of interest do not fully capture program impact in that year.

Thus, MSI's Impact 2 methodology allows organisations to move beyond cruder output measures to a better understanding of the role they play in the countries where they work.

Impact 2 also offers users the opportunity to model a wide range of results, of which percentage point contribution to increasing modern CPR is just one. While not discussed in this paper, Impact 2 is capable of producing a series of metrics related to the estimated health, demographic, and economic impacts of family planning services that are recognized as standards by the global health community. Such results are useful within many different contexts, including programme and policy advocacy, communications with governments and donors, and monitoring programme progress over time. Moreover, Impact 2 enables both retrospective and prospective modelling, a capability that empowers organisations with informed decision making and more realistic programme planning.

Impact 2 results can also play an important role in improving service delivery. The model structure and the estimates produced highlight the need for an organisation to carefully consider whom it is reaching (e.g. adopters, provider changers). Doing so ensures that its programme is playing a role in growing the market, not just increasing user numbers. As service providers answer the recent call to address the large unmet need for family planning worldwide, knowledge of how to accurately measure this expansion will be indispensable.

## List of abbreviations

CPR: contraceptive prevalence rate; WRA: women of reproductive age; LAPM: long-acting and permanent method; IUD: intrauterine device; MSI: Marie Stopes International; CYP: couple-year of protection; DALYs: disability-adjusted life years; PBI: previous birth interval; MMR: maternal mortality ratio; ANC: antenatal care; PAC: post abortion care; WHO: World Health Organisation; PSI: Population Services International; YLL: years of life lost; YLD: years of life lost to disability; TFR: total fertility rate; DHS: Demographic and Health Surveys; UN: United Nations; CCR: cumulative continuation rate; CD: Coale-Demeny; USAID: United States Agency for International Development.

## Competing interests

The authors declare that they have no competing interests.

## Authors' contributions

MBW led the development of the Impact 2 model, and wrote the first draft of this paper. KF provided technical review of the Impact 2 model and edited this paper. TB and KH conceptualised the original models, Impact 1.2 and REACH, which served as a foundation for Impact 2, and guided the development of the new model. All authors read and approved the final manuscript.

## Supplementary Material

Additional file 1 Service provision data from Marie Stopes Madagascar, 1996-2011, by numbers of commodities/services provided by method. These data show how many family planning commodities and services Marie Stopes Madagascar provided to clients from 1996-2011. They are organised by method. Click here for file

## Declarations

This article is part of the supplement of *BMC Public Health *Volume 13, Supplement 2, 2013: Use of health impact metrics for programmatic decision making in global health. Population Services International, a registered non-profit organization, provided the funding for the publication of this supplement. The full contents of the supplement are available online at http://www.biomedcentral.com/bmcpublichealth/supplements/13/S2.
