# Supplementary material for: Estimating the contribution of a service delivery organisation to the national modern contraceptive prevalence rate: Marie Stopes International's Impact 2 model
Source: BMC Public Health. 2013 Jun 17;13(Suppl 2):S5. doi: 10.1186/1471-2458-13-S2-S5 (PMC3684538; doi:10.1186/1471-2458-13-S2-S5)
Supplement: Additional file 1 — Service provision data from Marie Stopes Madagascar, 1996-2011, by numbers of commodities/services provided by method. These data show how many family planning commodities and services Marie Stopes Madagascar provided to clients from 1996-2011. They are organised by method. [file 1471-2458-13-S2-S5-S1.PDF]

Additional file 1: Service provision data from Marie Stopes Madagascar, 1996-2011, by numbers of commodities/services provided by method

	1996	1997	1998	1999	2000	2001	2002	2003	2004	2005	2006	2007	2008	2009	2010	2011
**Long-acting and permanent methods**																
Female sterilisation	272	329	390	469	588	299	131	578	1,557	3,699	5,252	7,275	7,083	8,607	12,015	8,605
Male sterilisation	18	43	52	50	44	41	16	32	100	136	210	276	188	226	223	254
5-year implants	0	0	0	0	490	166	429	470	799	1,216	1,972	3,125	6,206	17,535	26,899	17
3 year implants	0	0	0	0	0	0	0	0	0	0	0	0	0	0	0	34,158
10-year IUDs	507	722	672	674	811	628	566	512	610	561	1,021	1,299	1,305	7,321	20,264	24,077
**Short-term methods**																
Condoms					14,628	10,426	8,784	5,053	1,819	1,938	2,670	3,556	3,098	16,470	404,407	2,000,272
Pills (cycles)					0	0	0	0	0	34,812	33,706	40,447	31,708	25,680	32,146	36,381
Diaphragms					0	0	0	0	0	0	0	5	277	0	0	0
Foam tablets					0	0	0	0	0	0	0	5	277	0	0	0
3-month injectables					36,974	30,903	28,933	29,933	34,274	37,843	38,246	46,011	34,892	31,659	59,190	79,113
